# Sintilimab induced ICIAM in the treatment of advanced HCC: A case report and analysis of research progress

**DOI:** 10.3389/fimmu.2022.995121

**Published:** 2022-08-26

**Authors:** Hongxiang Ji, Zhijian Wen, Bin Liu, Hongbiao Chen, Qian Lin, Zhan Chen

**Affiliations:** Department of General Surgery, The Chenggong Hospital Affiliated to Xiamen University, Xiamen, China

**Keywords:** hepatocellular carcinoma (HCC), programmed cell death receptor 1/ligand 1 (PD-1/PD-L1), ICI-associated myocarditis (ICIAM), sintilimab, immune checkpoint inhibitor-associated adverse reactions (irAEs)

## Abstract

Immune checkpoint inhibitor-associated adverse reactions (irAEs) are a clinical treatment issue that requires additional attention when ICIs have significant survival benefits in patients with advanced hepatocellular carcinoma (HCC). Among them, ICIs-associated myocarditis (ICIAM) is a kind of severe irAE with a high mortality rate (17%–50%). Despite its low incidence (PD1/PD-L1 related: 0.41%–0.8%), ICIAM can significantly disturb the decision making of therapeutic schemes and even the survival outcomes of patients. ICIAM induced by sintilimab has not been reported in any complete clinical studies yet and understanding the clinical characteristics involved may inform better practices for the management. Here, we reported a 78 y/o patient with advanced HCC, who experienced ICIAM induced by sintilimab within a short course from treatment onset and found that adequate baseline examination before the implementation of the therapeutic scheme, regular monitoring of myocardial enzymonram and cardiac imaging were measures for the early detection, while glucocorticoid pulse therapy is still the best choice with timely and sufficient application. Simultaneously, the combination of other immunosuppressants may lead to better results. New-predictive markers and examination methods are still required to facilitate the early detection.

## Introduction

The application of immune checkpoint inhibitors (ICIs) has benefited the survival of patients with various kinds of advanced malignancies, such as non-small cell lung cancer (NCLC) and metastatic melanoma (1, 2). ICIs enhance the recognition of T cells by tumor cells by targeting the inhibition of cytotoxic T lymphocyte-associated protein 4 (CTLA-4) or programmed cell death receptor 1 (PD-1)/ligand 1 (PD-L1) to alter the immune escape microenvironment of tumors. In recent years, breakthroughs have also been made in the systematic treatment of advanced hepatocellular carcinoma (HCC) with the application of immunotherapy, which has broken the state of monotherapy with only tyrosine kinase inhibitors (TKIs) (3). The “Standard for the diagnosis and treatment of primary liver cancer (National Health Commission of the PRC 2022, China)” recommends that HCC patients in China with liver cancer staging (CNLC) IIb, IIIa, and IIIb have a choice of combined systemic therapy (4). Sintilimab (Tyvyt^®^) is the second immune checkpoint inhibitor independently developed and marketed in China in 2018 and was approved for systemic treatment of advanced HCC (the fourth indication) in 2021. The effectiveness of sintilimab combined with lenvatinib has been reported in many clinical studies (5). However, the lack of reports on severe ICI-associated myocarditis (ICIAM) events induced by sintilimab during HCC treatment led us to report a 78-y/o patient with advanced HCC who experienced ICIAM induced by sintilimab within a short course from treatment onset here. The treatment regime is 21 d per cycle, and the adverse reaction occurred on the first day of the second cycle. The patient, who had no history of heart disease, experienced acute myocardial injury involving the conduction system with insidious and rapid progression but no obvious symptoms at the beginning. Within 4 days, the patient developed a significant conduction block accompanied by abnormalities of the myocardial enzymonram. The adverse reaction was controlled after temporary cardiac pacemaker implantation and a high dose of methylprednisolone (HDMP) combined with gamma globulin therapy.

## Case presentation

**Figure 1:**
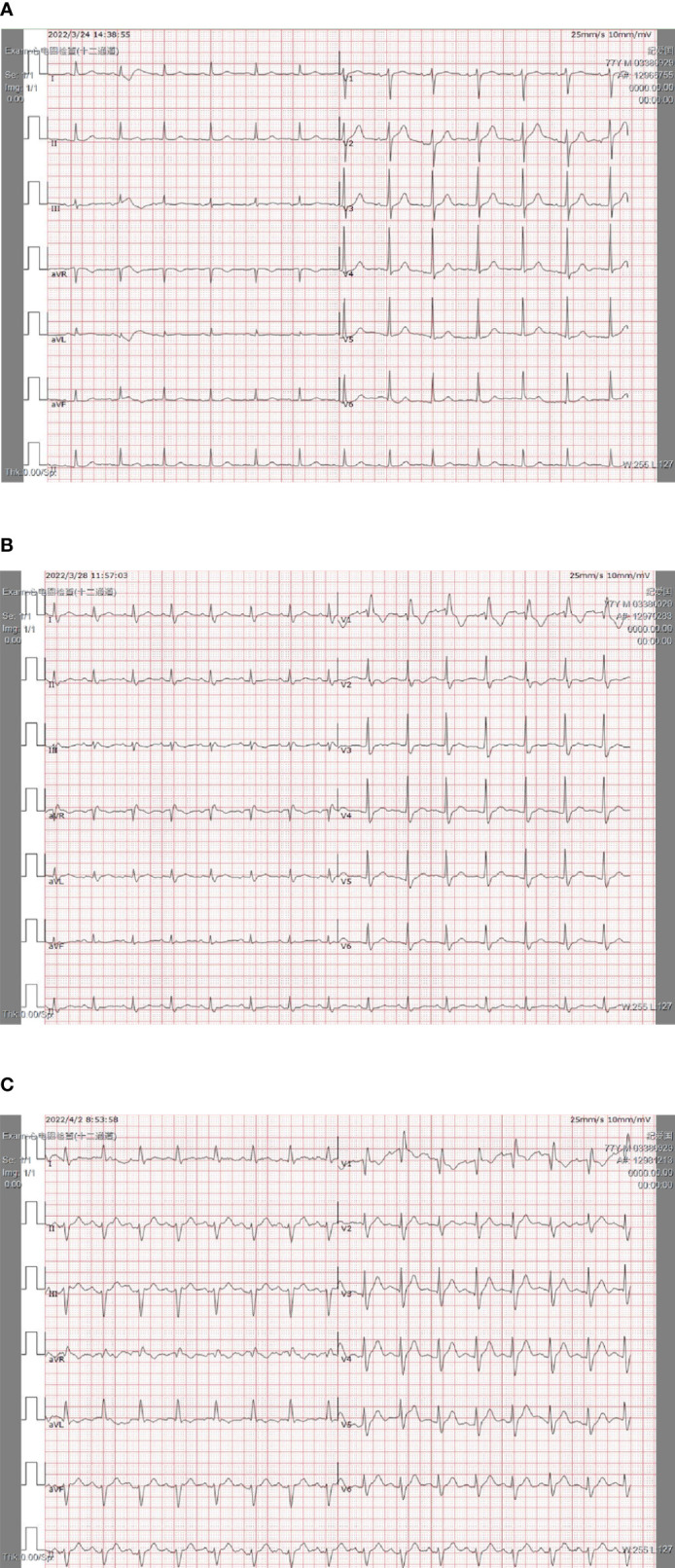
The rapid progress of the ECG of the patient’s within 4 days. **(A)** PI and II were upright; Pav was inverted. QRS widened equal to or longer than 0.12 s; V1rsR; QRS terminal in each lead was blunt and ST-T was changed secondary. **(B)** PI and II were upright; Pav was inverted; QRS was widened equal to or longer than 0.12 s; V1 rsR; II, III, aVF were changed to rS type; S III >S II; I, aVL leads were qR type; R avL >R I; QRS electrical axis was left biased. **(C)** Sinus heart rate; high atrioventricular block; abnormal Q-waves appeared in V2–V3 leads.

**Figure 2:**
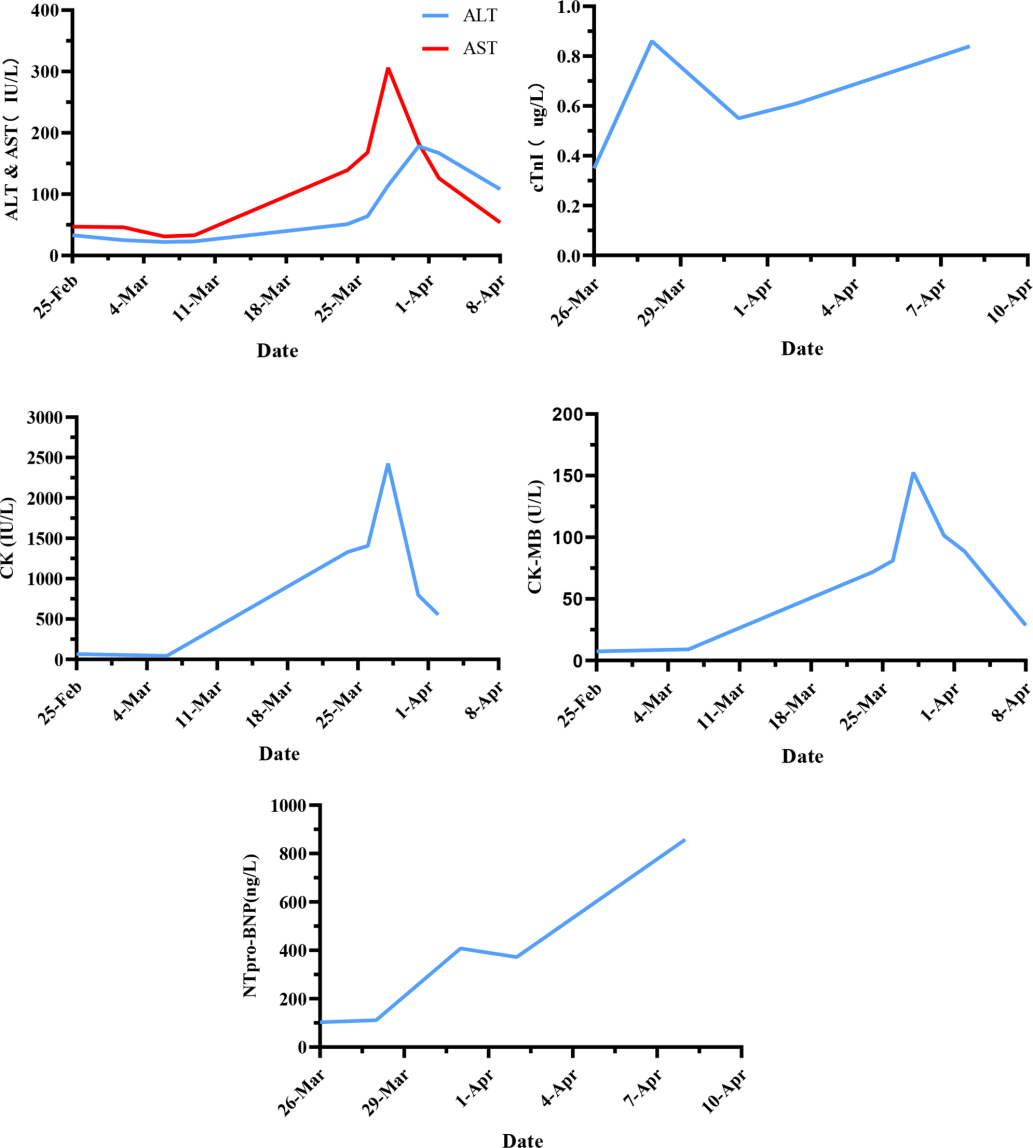
The changes in serum enzyme levels during the treatment (x-axis = date, y-axis = units symbols).

**Figure 3:**
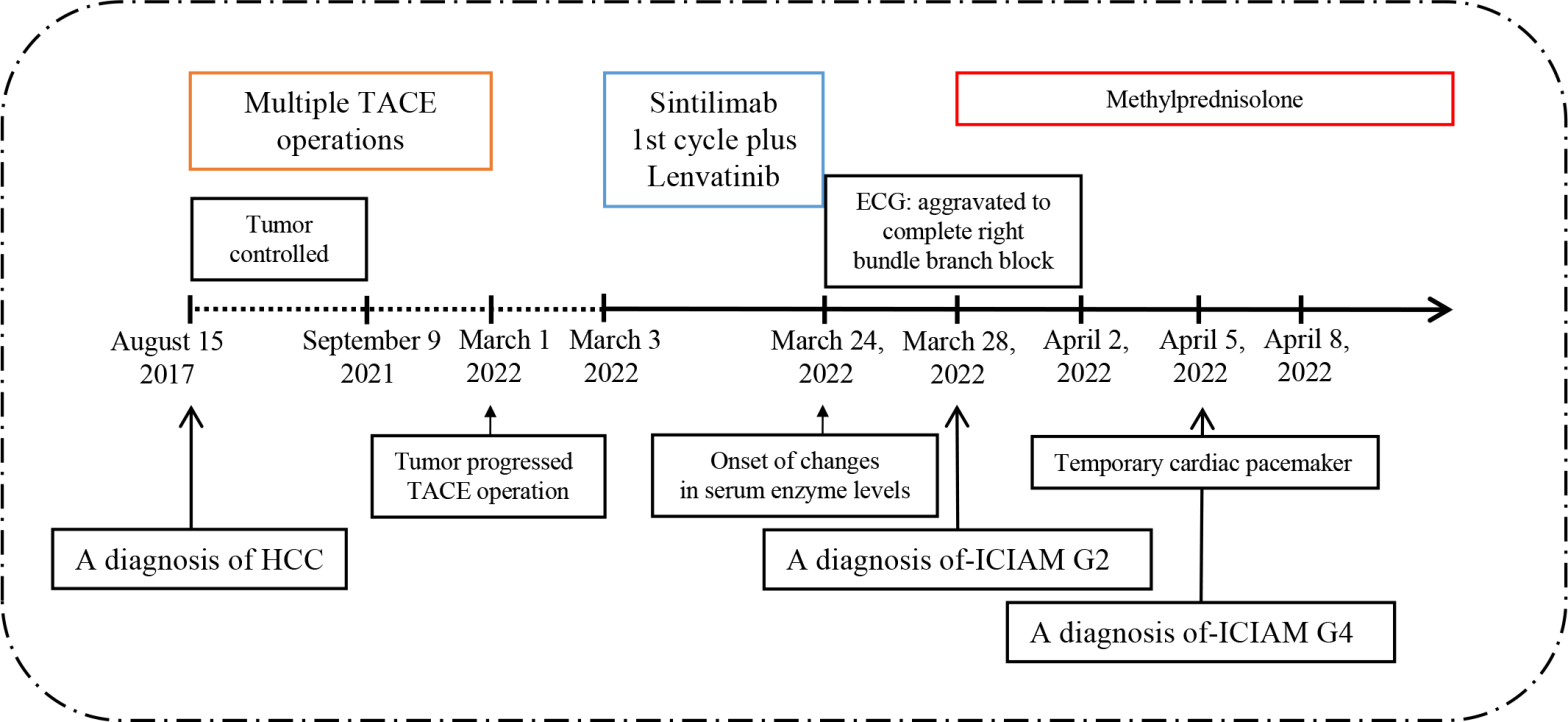
Timeline of disease diagnosis and treatment.

The patient was a 78-year-old male, weighing 80 kg, with a history of hypertension. He was admitted to the hospital on 15 August 2017 for transhepatic arterial chemotherapy and embolization (TACE) treatment (surgical operation was rejected for the risks associated) due to HCC (CNLC Stage IIa/Barcelona Clinic Liver Cancer, BCLC Stage B), and then received multiple TACE operations to control the tumor from 29 September 2017 to 9 September 2021. TACE was performed again due to the tumor progression on 1 March 2022, and a combined systemic therapy of sintilimab (200 mg Ivgtt 1/21 d) and lenvatinib (8 mg po 1/d) was planned. The therapeutic regimen was implemented on 3 March 2022. At that time, the baseline examination of the patient showed no obvious abnormalities in biochemical indicators, myocardial enzymonram, or electrocardiogram (ECG). Baseline monitoring was performed 1 week after the patient was safely discharged from the hospital after cycle 1, which showed no other changes except a mild liver function abnormality. When the patient planned to receive the second cycle of treatment on 24 March 2022, the baseline monitoring on admission showed abnormal elevation of creatine kinase (CK 1,330 IU/L) and creatine kinase-myocardial band (CK-MB 71.7 U/L). However, no abnormality was found in ECG or echocardiography. Therefore, the regimen was suspended for the consideration of safety, though the patient has not complained of any discomfort. After the cardiology consultation, it was suggested that additional measurements of troponin (cTnI), myoglobin (Mb), and BNP should be tested for further evaluation, and a 24-hour Holter examination should be performed for the differential diagnosis of non-ST elevation myocardial infarction (NSTEMI). On 28 March 2022, the CK (2,425 IU/L) and CK-MB (152.6 U/L) of the patient were further elevated with the abnormal of CTnI (0.86 ug/L) and Mb (>900 ug/L) at the same time. The ECG and the 24-hour Holter examination showed the complete right bundle branch block without significant ST-segment or T-wave abnormalities, and the patient still experienced no discomfort. So the diagnosis of the following: immune checkpoint inhibitor-associated adverse (irAE)-ICIAM G2 (Chinese Society of Clinical Oncology, CSCO, Management of immune checkpoint inhibitors-related toxicity 2021, China and CTCAE_4.03) was considered (6), and methylprednisolone was administered at 160 mg/d (2 mg/kg·d) for 3 days consecutively for treatment. After the condition of the patient improved, the dosage was reduced to 80 mg/d (1 mg/kg·d) from 1 April 2022. On 2 April 2022, the CTnI and NT-proBNP of the patient showed obvious rebounds. Simultaneously, the ECG suggested that the conduction block was aggravated and accompanied by the symptoms of fatigue and mild diarrhea. The patient was transferred to the cardiology hospital for further treatment immediately with a diagnosis of irAE-ICIAM G4 on 5 April 2022. On the day of transfer, a temporary cardiac pacemaker was implanted for the patient. On the third day after implantation, a 24-hour Holter examination was performed again, with the result still showing no abnormalities in ST-segment or T-wave. At the same time, the glucocorticoid therapy was adjusted to a pulse dose of methylprednisolone 1 g/d combined with gamma globulin 20 g/d for 3 consecutive days. The cardiac indicators and clinical symptoms of the patient improved significantly, with the ECG showing a turn back from III degree atrioventricular block to autonomic rhythm and the echocardiography showing no obvious abnormalities. Then, the standardized reduction of methylprednisolone was implemented (**Figures 1**–**3**).

## Discussion

ICIAM is a rare kind of irAEs which can manifest as explosive myocarditis, pericarditis, conduction block, heart failure, myocardial fibrosis, or acute coronary syndrome (ACS) and has a high mortality rate (7).

In several reports based on the World Health Organization (WHO) adverse drug reaction database (vigibase), the incidence of ICIAM is about 1.3%–5.8%, and the median time from the onset of ICI treatment to the occurrence of ICIAM is about 27–57 d, of which the incidence of ICIAM caused by PD-1/PD-L1 is about 0.41%–0.8%. In a retrospective analysis of 101 ICIAM cases, Moslehi et al. found that 64% of the patients experienced cardiotoxicity during the first or second cycle of ICI treatment, of which 76% occurred within the first 6 weeks from treatment onset, and 58 of the 101 patients were treated with PD-1 monotherapy (8–11). Another observational study from Jain et al. showed that conduction dysfunction accounted for 1.16% of ICIAM patients (12). The ORIENT-1 (NCT03114683) trial evaluated the safety and efficacy of sintilimab monotherapy among patients with relapsed or refractory classical Hodgkin’s lymphoma who had received at least second-line chemotherapy. One patient developed ICIAM (G3) and was forced to stop treatment. In 2019, Rong Peipei et al. reported a patient with thymoma who received sintilimab combined chemotherapy and developed ICIAM rapidly. The time of occurrence was only 13 days after the treatment onset, which may be the first case report in clinical of sintilimab related ICIAM in China. Huang et al. reported in a study that 48 patients with various advanced tumors were treated with sintilimab, and one of them developed ICIAM (G1) (13). Huan Bi et al. reported a case with lung squamous cell carcinoma (SCC) that developed ICIAM within 6 days plus three cycles after receiving sintilimab treatment (21 days/cycle) (14). Chen and Liang et al. reported the case of ICIAM in a chordoma patient who received sintilimab plus anlotinib treatment respectively (15, 16). The retrospective analysis of cases from 12 cancer treatment centers in China by Wang et al. showed that the incidence of ICIAM in China was about 1.05%, the median time of occurrence was 38 d, and 81.2% occurred in the first or second cycle of ICIs therapy with a high mortality rate. Among them, the incidence of ICIAM caused by PD-1/PD-L1 was about 0.6% (17).

At present, the time definition of irAEs can be divided into early onset (median onset time ≤2 months) and late onset (median onset time >2 months). For ICIAM, Salem et al. found that the incidence of ICIAM decreased significantly after 3 months of flat treatment (9). Considering the current situation, it seems necessary to adopt a separate name and time definition for the serious and rapid-progressing style of irAEs, including ICIAM, and corresponding inclusion criteria should be formulated, so as to facilitate the accumulation of relevant cases and coping experience in each clinical center.

Before the implementation of ICIs treatment, the assessment of the baseline status of the patients is so important that for HCC patients the assessment should be very carefully as their liver function may fluctuate abnormally due to basic diseases (liver cirrhosis), tumor progression or other treatment interventions. In particular, it is not easy to determine the primary injured organs only based on the fluctuation of AST or CK in peripheral blood samples from an HCC patient. If there are no obvious clinical manifestations, the significant increase of CK or CK-MB compared to baseline data requires clinicians to be highly vigilant against the early occurrence of ICIAM. The early detection of ICIAM also requires attention to capture non-specific clinical manifestations in combination with the monitoring of ECG, myocardial enzymonram, echocardiography, or cardiac magnetic resonance. The retrospective study from Mahmood et al. showed that the abnormal rate of troponin, pro-BNP, and ECG in patients with ICIAM was 94%, 66%, and 89%, respectively, while the variation rate of left ventricular ejection fraction was only 49% (8). How to detect cardiac injury before the dysfunction of the left ventricular? A single center study by Zhao et al. showed that the global longitudinal strain (GLS) reduction may be an independent risk factor for ICIAM, which could be assessed early by using cardiovascular magnetic resonance feature tracing (CMR-FT) technology and could indicate cardiac injury prior to the onset of severe left ventricular dysfunction (18). Yu et al. found that the right ventricular systolic function often declined after PD-1/PD-L1 inhibitor treatment, and the decline mostly occurred during 21–42 d. This change can be detected early by using the two-dimensional speck tracing technique of echocardiography on the plane displacement of the tricuspid annulus during systole. In terms of research progress in predicting markers, studies have shown that soluble growth stimulating gene 2 protein (sST2) is a good marker of explosive myocarditis (19), which may be used to predict the prognosis of patients with ICIAM. In a meta-analysis of biomarkers for the detection of cardiac function disorders related to tumor therapy by Xiao et al., it was shown that the C-reactive protein (CRP), in addition to CTn and BNP, can also be an applicable biomarker for the occurrence of ICIAM (20). The upregulation of the CXC chemokine family, which is related to T-cell activation in the treatment of ICIs, may also suggest the occurrence of ICIAM (21, 22). Interestingly, John et al. reported three cases of ICIAM patients with diplopia and ptosis as the first clinical symptoms and suggested that clinicians should be alert to the possibility of ICIAM when patients appeared to have ocular symptoms (23). Presently, the “Chinese expert consensus on the surveillance and management of immune checkpoint inhibitor-related myocarditis” (2020 version) has recommended the classification and monitoring methods of ICIAM, which has an important reference value (24).

The first choice for coping with ICIAM is still impulse therapy with glucocorticoids. Mahmood et al. found that the initial high-dose of glucocorticoids would be more effective for patients, which can reduce the incidence of major adverse cardiac events (MACE) (8). Ma et al. reported eight cancer patients, including one HCC, who developed severe ocular symptoms during ICI treatment. These symptoms were controlled by systemic or topical glucocorticoid therapy, which indicates that adequate treatment of glucocorticoids also plays a key part in the treatment of other types of irAEs (25). However, pretreatment of irAEs by using glucocorticoids is still not recommended because of the potential reduction in anti-tumor efficacy from ICIs, unless the patient has specific indications (e.g., infusion response or concurrent chemotherapy). If the condition of patients with mild myocarditis still deteriorates to a severe or critical type after receiving a routine dose, such as this patient, the glucocorticoids should be adjusted to the pulse dose without delay. In addition to glucocorticoids, there is no relevant research suggesting a better combination scheme. The selection of additional drugs may be based on the drug availability of medical institutions and the changes in the course of ICIAM. Many guidelines have recommended that if the condition of patients with severe myocarditis has not improved after receiving a pulse dose of glucocorticoids for 24 h, clinicians should apply one to two additional drugs jointly or sequentially, the drug choices including intravenous immunogloblin (IVIG), anti-thymocyte globulin (ATG), mycophenolate mofetil, tacrolimus, or infliximab (dose >5 mg/kg is forbidden for moderate to severe heart failure) (26, 27).

As a new immunotherapeutic drug for advanced HCC, sintilimab is being widely used in domestic tumor centers now due to its good efficacy, easy accessibility, and economic advantages. However, irAEs caused by sintilimab, especially ICIAM, are not completely controllable. Regular monitoring in the process of treatment for early diagnosis and the more aggressive application of glucocorticoids are preferred methods for coping with sintilimab-related ICIAM. Meanwhile, further experience needs to be accumulated from more clinical cases.

## Data availability statement

The original contributions presented in the study are included in the article/supplementary material. Further inquiries can be directed to the corresponding author.

## Ethics statement

Written informed consent was obtained from the individual(s) for the publication of any potentially identifiable images or data included in this article.

## Author contributions

HJ and ZW contributed equally to this work. ZW provided case information and contributed to manuscript revision. HJ managed data analysis and drafted the manuscript. BL, HC, and QL performed the clinical management of the patient. All authors contributed to the article and approved the submitted version.

## Funding

This work was supported by the Key Laboratory of Biliary Tract Diseases of Xiamen and the Xiamen Medical and Health Guidance Project in 2021 (Approval number: 3502z20214zd1187).

## Conflict of interest

The authors declare that the research was conducted in the absence of any commercial or financial relationships that could be construed as a potential conflict of interest.

## Publisher’s note

All claims expressed in this article are solely those of the authors and do not necessarily represent those of their affiliated organizations, or those of the publisher, the editors and the reviewers. Any product that may be evaluated in this article, or claim that may be made by its manufacturer, is not guaranteed or endorsed by the publisher.
